# Supplementary material for: Individualized Fortification Based on Measured Macronutrient Content of Human Milk Improves Growth and Body Composition in Infants Born Less than 33 Weeks: A Mixed-Cohort Study
Source: Nutrients. 2023 Mar 22;15(6):1533. doi: 10.3390/nu15061533 (PMC10052754; doi:10.3390/nu15061533)
Supplement: Supplementary file 1 [file nutrients-15-01533-s001.zip › nutrients-2201556-supplementary.pdf]

Supplementary Table S1. Comparison between enrolled and not enrolled eligible infants.

Eligible = 275	Enrolled	Not enrolled	*p*-value
	185	90	
Gestational age, weeks; mean (SD)	29.6 (2,19)	29.6 (2.52)	0.567**
Birth weight z-score; median (P_{25}-P_{75})	-0.10 (-0.74-0.28)	-0.31 (-0.85-0.35)	0.489**
Twins; n (%)	56 (30.3)	34 (37.8)	0.213***
Prenatal steroids; n (%)	175 (94.6)	85 (94.4)	1.000*
SNAPPE II; median (P_{25}-P_{75})	13 (0-25)	15 (5-30)	0.269**
Extreme preterm; n (%)	40 (21.6)	25 (27.8)	0.260***

*Fisher'exact test; ** Mann-Whitney U; *** Chi-Square test

Supplementary Table S2. Comparison between infants completing the exposure period of the study (N=185) and those lost to follow-up.

	Completed exposure period of the study	Lost to follow-up	*p*-value
	115	70	
Gestational age, weeks; mean (SD)	29.6 (2.15)	29.5 (2.27)	0.894*
Birth weight z-score; median (P_{25}-P_{75})	-0.03 (-0.62-0.31)	-0.35 (-0.82-0.20)	0.171*
Twins; n (%)	39 (33.9)	17 (24.3)	0.167**
Extreme preterm; n (%)	26 (22.6)	14 (20.0)	0.338**
SNAPPE II; median (P_{25}-P_{75})	10 (0-24)	15 (7-29)	0.031*
Prenatal steroids; n (%)	108 (93.9)	67 (95.7)	0.316***
Postnatal steroids; n (%)	5 (4.3)	3 (5.7)	0.642***
Late-onset sepsis; n (%)	37 (32.2)	15 (21.4)	0.057**
Necrotizing enterocolitis III; n (%)	0	2 (2.9)	0.142***
Intraventricular hemorrhage IV; n (%)	2 (1.7)	4 (5.7)	0.147***
Bronchopulmonary dysplasia; n (%)	10 (8.7)	5 (7.1)	0.354**
Hospital stay, days; median (P_{25}-P_{75})	46 (34-64)	53 (29.5-71)	0.531*

* Mann-Whitney U test; ** Chi-Square test; *** Fisher'exact test

Supplementary Table S3. Comparison between children born at gestational less than 28 weeks and greater than or equal to 28 weeks.

	gestational age at birth < 28 weeks	gestational age at birth ≥ 28 weeks	*p*-value
	26	89	
SNAPPE II; median (P_{25};P_{75})	20 (9;30)	13 (0;23)	**0.033**
Late-onset sepsis; n (%)	18 (69.2)	19 (21.3)	0.003**
Necrotizing enterocolitis III; n (%)	0	0	-
Intraventricular hemorrhage IV; n (%)	1 (3.8)	1 (1.1)	0.410*
Bronchopulmonary dysplasia; n (%)	6 (23.1)	4 (4.5)	0.018*
Hospital stay, days; median (P_{25};P_{75})	71 (63;99)	40 (31;53)	<0,001

Mann-Whitney test; *Fisher'exact test; ** Chi-Square test

Supplementary Table S4. Comparison between groups, of infants' age before, during, and after exposure, time intervals of exposure, and percentage of exposure days during enteral feeding and hospital stay. Group 1— fortified HM based on its assumed macronutrient content. Group 2— fortified HM based on its measured macronutrient content.

	Group 1<br>N=57	Group 2<br>N=58	*p*-value
Gestational age (wks) at beginning of the exposure, in weeks; mean (SD)	31.5 (1.975)	31.45 (2.01)	0.412
Age at beginning of exposure, in days; median (P_{25};$P7_{5}$)	11 (9;13)	11 (8;14)	0.721
Postmenstrual age at the end of exposure, in weeks; mean (SD)	31.1 (1.98)	31.5 (2.01)	0.412
Postmenstrual age at hospital discharge, in weeks; mean (SD)	36.2 (1.21)	37.0 (2.40)	0.082
Time interval before exposure (days); median (P_{25};P_{75})	10.0 (9.0-12.0)	11.0 (8.0-14.0)	0.126
Time interval during exposure (days); median (P_{25};P_{75})	28.0 (17.5-50.0)	23.0 (16.0-36.0)	0.072
Time interval from end of exposure and discharge (days); median (P_{25};P_{75})	7.0 (3.0-12.0)	9.5 (5.0 19,3)	0.191
Percentage of exposure days during enteral feeding; mean (SD)	60.2 (20.83)	56.3 (18.82)	0.300
Percentage of exposure days during hospital stay; mean (SD)	53.4 (19.89)	50.9 (17.02)	0.588

SD – standard deviation; Student-t test, Chi-Square test, Fisher's exact test, median test, or Mann-Whitney test as appropriate

Supplementary Table S5. Proportions of exposure days in which the intake of energy, P/E, and macronutrients did not reach the minimum recommended or exceeded the maximum recommended intakes (N=115). Group 1— fortified HM based on its assumed macronutrient content. Group 2— fortified HM based on its measured macronutrient content.

	Recommended by ESPGHAN 2010*	Group 1	Group 2	*p-value*
		N=57	N=58	
Energy intake, kcal/kg/d	110-135			
Proportion of days (%) in which the energy intake did not reach the minimum recommended		32.6	12.1	**<0.001**
Proportion of days (%) in which the energy intake exceeded the maximum recommended		25.8	30.2	**0.002**
Protein intake, g/kg/d	< 1 Kg body weight: 4.0-4.5<br>1-1.8 Kg body weight: 3.5-4.0			
Proportion of days (%) in which protein intake did not reach the minimum recommended	<1 kg body weight	31.3	31.4	0.494*
	≥ 1 kg body weight	25.0	15.5	0.047*
Proportion of days (%) in which the protein intake exceeded the maximum recommended	<1 kg body weight	51.5	46.4	0.235*
	≥ 1 kg body weight	51.9	54.2	0.372*
Protein-to energy ratio	< 1 Kg body weight: 3.6-4.1<br>1-1.8 g body weight: 3.2-3.6			
Proportion of days (%) in which the protein-to energy ratio intake did	<1 kg body weight	34.3	1.3	**< 0.001**

not reach the minimum recommended	≥ 1 kg body weight	27.8	0	**< 0.001**
Proportion of days (%) in which the protein-to energy ratio intake exceeded the maximum recommended	<1 kg body weight	54.5	95.4	**< 0.001**
	≥ 1 kg body weight	56.8	99.3	**< 0.001**
Fat intake, g/kg/d	4.8-6.6 g/kg/d			
Proportion of days (%) in which the fat intake did not reach the minimum recommended		37.6	20.2	**<0.001**
Proportion of days (%) in which the fat intake exceeded the maximum recommended		23.3	46.5	**<0.001**
Carbohydrate intake, g/kg/d	11.6-13.2 g/kg/d			
Proportion of days (%) in which carbohydrate intake did not reach the minimum recommended		37.6	20.2	<0.001
Proportion of days (%) in which the carbohydrate intake exceeded the maximum recommended		23.3	46.5	<0.001

Chi-Square test

* Agostoni C, Buonocore G, Carnielli VP, et al. Enteral nutrient supply for preterm infants: Commentary from the European Society of Paediatric Gastroenterology, Hepatology and Nutrition Committee on Nutrition. J Pediatr Gastroenterol Nutr. 2010;50(1):85-91. doi:10.1097/MPG.0b013e3181adaee0
